# What is Bottom-Up and What is Top-Down in Predictive Coding?

**DOI:** 10.3389/fpsyg.2013.00276

**Published:** 2013-05-17

**Authors:** Karsten Rauss, Gilles Pourtois

**Affiliations:** ^1^Institute of Medical Psychology and Behavioral Neurobiology, Faculty of Medicine, University of TübingenTübingen, Germany; ^2^Psychopathology and Affective Neuroscience Lab, Department of Experimental Clinical & Health Psychology, Ghent UniversityGhent, Belgium

**Keywords:** bottom-up, predictive coding, top-down, V1, vision

## Abstract

Everyone knows what bottom-up is, and how it is different from top-down. At least one is tempted to think so, given that both terms are ubiquitously used, but only rarely defined in the psychology and neuroscience literature. In this review, we highlight the problems and limitations of our current understanding of bottom-up and top-down processes, and we propose a reformulation of this distinction in terms of predictive coding.

## Introduction

A central assumption in predictive-coding theories is that activity in the nervous system reflects a process of matching internally generated predictions to external stimulation (Heekeren et al., 2008; Bar, 2009). Evidence for this assumption has been collected at different levels of neural processing (Rao and Ballard, 1999; Hosoya et al., 2005; Muckli et al., 2005; Summerfield et al., 2006; Alink et al., 2010), which suggests that predictive-coding operates across a wide range of spatial and temporal scales.

A second important assumption in predictive coding is that predictions are transferred from hierarchically higher levels of processing to lower ones, whereas signals traveling in the opposite direction encode prediction errors (Rao and Ballard, 1999; Serences, 2008; Friston, 2009; Grossberg, 2009). In accordance with terminology commonly used in research on perception, it has thus been suggested that predictive signaling reflects top-down processes, whereas prediction-error signaling constitutes bottom-up processing (Friston, 2009; Alink et al., 2010; Hesselmann et al., 2010).

In this review, we argue that predictive coding provides a powerful conceptual framework that goes beyond the standard dichotomy of “bottom-up” and “top-down.” We first provide an overview of previous attempts at defining bottom-up and top-down processes, and we highlight their problems and limitations. We then outline how predictive coding offers a unique perspective for re-defining what is meant by these terms.

Throughout the remainder of this paper, we refer to *ascending* and *descending* connections when discussing the anatomy of biological systems or the architecture of artificial ones (Friston, 2005; Clark, in press). Functional activity along these two types of connections is referred to as *feedforward* and *feedback*, respectively. We avoid additional terms such as “recurrent” or “reentrant,” because we believe they can be subsumed under “feedback” in most cases.

## A Short History of Bottom-Up and Top-Down

More than 30 years ago, Kinchla and Wolfe (1979) set out to test whether visual processing is organized bottom-up or top-down. Following up on earlier work (Reicher, 1969; Navon, 1977), the authors used compound stimuli, where a global shape is made up of smaller, local elements. Upper-case letters were used as stimuli, and top-down processing was assumed to manifest itself in shorter response times (RTs) if the global shape, rather than the local elements, corresponded to a target letter. Bottom-up processing was defined as “the opposite,” i.e., faster responses if the local stimulus elements, rather than the overall shape, corresponded to a target letter. Results indicated that whether a global shape or its constituent elements are processed faster critically depends on stimulus size. Kinchla and Wolfe (1979) concluded that visual perception does not proceed strictly bottom-up or top-down, but “middle-out.” They suggested that the visual system uses the information most readily available in the context of, for example, a particular stimulus size and viewing distance. Based on this information, the system would then work its way toward more global or more local aspects, as required by the task.

More recently, Melloni et al. (2012) examined the generation of saliency maps in the visual cortex with functional magnetic resonance imaging (fMRI). The authors refer to bottom-up salience as the degree of difference between a stimulus and its neighbors. In their visual search task, target and distractor gratings differed in orientation. Bottom-up salience was manipulated by adding color as an additional stimulus dimension. Thus, the target stimulus could be either a singleton in terms of color and orientation; a singleton in terms of orientation only; or a singleton in terms of orientation accompanied by a distractor singleton in terms of color. Top-down control is described by Melloni et al. (2012) as the influence of our inner goals on stimulus selection. This was manipulated by presenting trials of different degrees of bottom-up salience either in blocks or randomly intermixed. Results indicated that primary visual cortex (V1) encodes only bottom-up salience, V2 encodes only top-down control settings, and V4 encodes the interaction between the two. The authors concluded that multiple saliency maps are present at different levels of processing. Stimulus selection could thus be flexibly adapted by referring to the saliency map most relevant in the context of a given task (Weidner et al., 2009).

In the three decades between the studies of Kinchla and Wolfe (1979) and Melloni et al. (2012), conceptual and methodological advances have substantially increased our knowledge of sensory processing in general and of the visual system in particular. What hasn’t changed during this time is our notion of bottom-up and top-down processes. Staying with our first example, Kinchla and Wolfe (1979) wrote:

*[*…*] the electrophysiological analysis of “receptive fields” seemed to suggest a bottom-up mode of processing: Cells associated with progressively more complex fields [*…*] were found as one went from the retina to the visual cortex, as if systems for detecting low-order “features” [*…*] fed into systems for detecting progressively more complex patterns. (Kinchla and Wolfe, 1979, p. 225)*

*[*…*] it has been suggested that the order of visual processing is best described as a top-down process, with higher-order forms processed first, followed by lower-order forms [*…*] (ibd.)*

Compare this to a more recent definition is given by Palmer (1999):

*“Bottom-up” processing [*…*] refers to processes that take a “lower-level” representation as input and create or modify a “higher-level” representation as output. Top-down processing [*…*] refers to processes that operate in the opposite direction, taking a “higher-level” representation as input and producing or modifying a “lower-level” representation as output. (Palmer, 1999, pp. 84–85)*

The common denominator of these and virtually all other definitions of bottom-up and top-down processes can be summarized as follows:

1. Information processing is organized hierarchically.
2. Lower levels of the hierarchy represent detailed stimulus information, while higher levels represent more integrated information.
3. Information exchange between levels is bidirectional.

The apparent simplicity of these assumptions may explain why the terms bottom-up and top-down are used so frequently in research on perception. However, these assumptions can be questioned, and we briefly highlight some of the problems surrounding them.

### A note on hierarchies

Felleman and Van Essen (1991) advocated a distributed processing hierarchy in the primate visual cortex based on anatomical connectivity patterns. More recently, however, Hegdé and Felleman (2007) noted that a hierarchically organized anatomical structure does not imply that visual processing is itself hierarchical, nor that the functional hierarchy (if it exists) matches the anatomical one (Rousselet et al., 2004). Moreover, Hegdé and Felleman (2007) state that the anatomical hierarchy itself is clear-cut only up to the level of areas V4 and MT, with the sender-receiver distinction becoming less evident at higher levels. Thus, the notion of hierarchical processing can be criticized on both structural and functional grounds.

These criticisms may be partly resolved by adopting a more flexible view of structural and functional hierarchies. An example for such a view is provided by Engel et al. (2001), who distinguish four “flavors” of top-down:

1. an *anatomical* one, equating top-down processes with functional activity along descending connections between the levels of the hierarchy
2. a *cognitivist* one, where top-down means hypothesis-driven processing
3. a *gestaltist* one, viewing top-down processes in terms of contextual modulations of bottom-up processing
4. a *dynamicist* one, describing top-down processes in terms of an entrainment of local neuronal populations by widespread oscillatory activity in distant and distributed brain regions.

The last of these flavors does not require a fixed anatomical or functional hierarchy. It accommodates flexible recruitment of brain regions for different tasks, without negating the known specializations of these regions.

Indeed, the difficulties in establishing clear-cut processing hierarchies in the central nervous system based on either anatomical or functional criteria may be a direct consequence of the system’s adaptability. Thus, rather than relying on a fixed hierarchy for processing all types of stimuli and performing all sorts of tasks, it seems likely that the system uses different hierarchies for different tasks. This does not mean that the notion of processing hierarchies is obsolete, but that hierarchical processing should be seen as a conceptual simplification.

Incidentally, the problems associated with hierarchical processing models were already recognized by Kinchla and Wolfe (1979). They stated that although individual images could usefully be described in terms of a hierarchy of features, the knowledge underlying our perception of these images had neither top nor bottom. Nevertheless, they argued that conceptualizing this knowledge as hierarchically organized remains a useful simplification. Intriguingly, their discussion also points to the role of predictability in establishing these hierarchies:

*[*…*] a major reason for such [a hypothetical hierarchical] organization is the life-long sequential pattern of our visual experience whereby recognition of a form at one level of structure is an almost invariant precursor of the recognition of forms at levels slightly higher or lower. (Kinchla and Wolfe, 1979, p. 229)*

## Problems

Even if one accepts the basic assumptions underlying hierarchical processing models, there are a number of problems associated with current notions of bottom-up and top-down processes. Some examples from the literature will demonstrate that one author’s top-down may well be another one’s bottom-up.

### A fuzzy dichotomy

In a recent position paper, Theeuwes (2010a) reviewed evidence for the notion that initial processing of a visual stimulus is exclusively driven by bottom-up factors. The author focuses on selection processes in visual search tasks and defines top-down selection as:

completely under control of the intentions of the observer

an active volitional process

based on expectancy and goal set (Theeuwes, 2010a, pp. 77–78)

Bottom-up selection, on the other hand, is described as:

determined by the feature properties present in the environment

passive [and] automatic

associated with salience

driven by emotional content or previous experience (ibd.)

Independent of whether or not one subscribes to Theeuwes’ (2010a) point of view, the number of concepts invoked to define two commonly used terms suggests that there is actually more to consider than a simple dichotomy. In other words, the use of a simple binary classification does not appear to capture the profusion of mental processes or functions described in these statements, which include intention, volition, expectation, and emotion. Accordingly, a large part of the discussion of Theeuwes’ (2010a) target paper concerns the delimitation of bottom-up and top-down processes (Egeth et al., 2010; Folk and Remington, 2010; Kristjánsson, 2010; Theeuwes, 2010b).

### Searching for the top

Importantly, the problems with distinguishing bottom-up from top-down processes are not limited to psychological studies. Confusion also seems to reign at the apparently more solid level of cellular neuroscience.

For example, Roland et al. (2006) used voltage-sensitive dye (VSD) imaging in anesthetized ferrets. The authors found that exposure to a luminance-defined square elicited forward and lateral activation spread that was followed by a feedback wave of activity traveling from extrastriate visual areas to V1. This feedback wave selectively highlighted first the stimulus representation and then the representation of the background. Roland et al. describe this feedback wave as a possible mechanism of top-down influences on early visual cortex.

Kuhn et al. (2008), on the other hand, used depth-resolved VSD imaging in mice during anesthesia and wakefulness. The authors observed characteristic desynchronizations in layer 1 of the somatosensory cortex upon awakening. They consider this as evidence for long-range cortical and thalamic input exerting top-down control over sensory processing during wakefulness.

Arguably, the term “top-down” is conceptually useless if it describes neural activity during both wakefulness (Kuhn et al., 2008) and anesthesia (Roland et al., 2006), with sources in either closely neighboring regions (Roland et al., 2006) or anywhere in the nervous system (Kuhn et al., 2008). However, such an interpretation directly emerges from what Engel et al. (2001) termed the anatomical flavor of top-down. That is, if activity along descending fibers necessarily reflects top-down processes, the latter may originate from any source connected to the current region of interest, provided that the source is located at a higher level of the hierarchy. And if source activity persists during sleep or anesthesia, this would also qualify as top-down activity.

Barlow (1997) succinctly noted this problem, stating that the visual system has no top. Reviewing potential sources of knowledge used in visual processing, he argues that the interaction of the genetically determined structure of the visual system and the redundancies present in all visual images may explain many of the effects usually attributed to top-down factors. He concludes:

*To avoid the top-down/bottom-up dichotomy blinding us to more important questions, the term “top-down” should perhaps be challenged whenever it is used [*…*] (Barlow, 1997, p. 1146)*

Barlow’s (1997) understanding of top-down processes is that they involve outside knowledge previously acquired through training or teaching and brought to act upon current sensory input. Here, “outside” refers to regions of the nervous system not primarily concerned with visual processing.

While we fully agree with Barlow’s (1997) conclusion, it seems what his argument essentially highlights is the vagueness of what is meant by a top-down process. His critique rests on the assumption that bottom-up processing consists of the interplay between the system’s genetically predetermined structure and the organism’s current environment. However, since the structure of the system also defines how it stores and retrieves previously encoded information, his understanding of bottom-up processes seems to leave hardly any room for top-down processes.

### Bottom-up/top-down vs. feedforward/feedback

In summary, current notions of bottom-up and top-down processes lump together largely disparate structures and functions. As a consequence, it is often unclear whether there is a difference between bottom-up and top-down processes on the one hand and feedforward and feedback activity, on the other. In this context, it is interesting to note that a number of authors speak of “top-down feedback” to refer to attention-induced modulations of either stimulus-evoked activity (Martínez et al., 2001), baseline activity (Kastner et al., 1999), or background connectivity (Al-Aidroos et al., 2012). In most of these cases, the aim seems to be to highlight the parallels between the psychological description of higher cognitive processes and the anatomical description of fibers connecting high-level to low-level regions. However, a more literal interpretation would suggest that, if one has to specify that a certain type of feedback is top-down, other types of feedback are not. We will return to this idea in the following section.

### Plasticity

In an original demonstration of how culture shapes perception, Tse and Cavanagh (2000) showed that the direction of apparent-motion perceived during the stepwise presentation of a Chinese character differs between Chinese and American participants. The character in question was unveiled one stroke at a time, without actual movements contained in the stimulus. Based on Gestalt principles (Wagemans et al., 2012a,b), the last stroke was expected to be perceived as a right-to-left movement, and this is what American subjects reported seeing. On the other hand, a majority of Chinese subjects reported seeing a movement from left to right, in accordance with the movement performed when writing the character. The authors concluded that bottom-up cues for motion perception can be overridden by top-down factors linked to culturally shaped expectations.

In their discussion, Tse and Cavanagh (2000) note that:

The “standard” view of top-down processing is that later visual areas influence earlier areas via feedback connections. There are other possibilities, however. Expectation and knowledge could in principle alter the circuitry involved in grouping, in which case a top-down influence would be exerted in a bottom-up manner (Tse and Cavanagh, 2000, p. B32)

This interpretation of top-down processes raises an important issue: beyond the differences in how bottom-up and top-down processes are conceptualized, individual differences may well lead to situations where one participant’s bottom-up is another one’s top-down.

## Solutions

Given these ubiquitous problems, we believe it is time that the concepts of bottom-up and top-down be refined based on current evidence. We briefly highlight previous work aiming in this direction, before outlining a predictive-coding model that may help resolve some of the issues outlined above.

### Top-down ≠ feedback

In an insightful commentary on Theeuwes (2010a), Rauschenberger (2010) argues that the dichotomy between bottom-up and top-down processes should be abandoned altogether in light of ever-increasing evidence on the importance of interactive information processing in perception (Di Lollo et al., 2000). Rauschenberger (2010) goes on to propose that the directionality of neural pathways does not necessarily correspond to their primary or exclusive involvement in bottom-up or top-down processes.

The idea that ascending and descending pathways may be jointly involved in both bottom-up and top-down processes seems to us the single most important insight for salvaging these concepts. Accordingly, this idea is at the core of our own proposals below.

### A third element

Following the lively discussion surrounding his position paper (Theeuwes, 2010a), Theeuwes co-authored an article entitled “Top-down vs. bottom-up attentional control: a failed theoretical dichotomy” (Awh et al., 2012). The authors make the point that attentional control cannot be fully described when only bottom-up salience and top-down goals are taken into account. Rather, selection history needs to be considered as well, i.e., whether a particular stimulus was previously task-relevant or rewarded. Awh et al. (2012) highlight how differences in selection history are often confounded with top-down effects such as selective attention, and how this may explain some of the contradictory findings in the visual search literature (e.g., Maljkovic and Nakayama, 1996; Wolfe et al., 2003).

The central and sometimes paradoxical role of selection history on attentional control is also in accordance with the idea that active predictions of incoming information may affect sensory processing at multiple levels. As Awh et al. put it:

*[*…*] we believe that [selection history effects] share one core feature: in each case, past selection episodes are recapitulated in subsequent trials when the relevant context is encountered again. (Awh et al., 2012, p. 440)*

Notwithstanding the importance of selection history effects, we are unsure whether adding a third concept to the discussion is helpful. Indeed, the definition given by Awh and co-workers is so broad that it spans virtually all types of neural activity, as selection history may be encoded at very different hierarchical levels (Treue, 2003) and on very different time-scales (Barlow, 1997).

### A predictive-coding account

In the following, we outline a predictive-coding model from which we derive simple and unequivocal definitions of bottom-up and top-down processes. Our goal is not to provide detailed guidelines for distinguishing these processes in complex biological systems. Rather, we aim to re-establish top-down and bottom-up as useful heuristic categories. In order to do so, we start from two simple premises:

1. Bottom-up and top-down are not opposites.
2. Within hierarchical systems, both ascending and descending connections are involved in bottom-up and top-down processes.

These premises are an extension of the main idea underlying predictive-coding theories and more general accounts of brain function (Summerfield and Egner, 2009; Friston, 2010): namely, that bidirectional information exchange between the levels of hierarchical systems serves to reconcile incoming information with internally generated predictions. If this is the case, there are two classes of questions one may ask about the system:

1. How is stimulus information compared to predictions?
2. Where do predictions come from, and where are prediction errors routed to?

For the second class of questions, we believe the most interesting functional unit is the loop formed by a central region that generates predictions and a lower-level region that receives stimulus information and/or prediction-error signals from the periphery.

Now consider a biologically inspired, yet-to-be programed computer model similar to previously published computational accounts of the visual cortex (Rao and Ballard, 1999). The model system is hierarchically organized over several levels, with increasingly complex stimulus attributes represented at higher levels. For the time being, we further assume that ascending and descending connections only link neighboring areas within the hierarchy.

**Figure 1:**
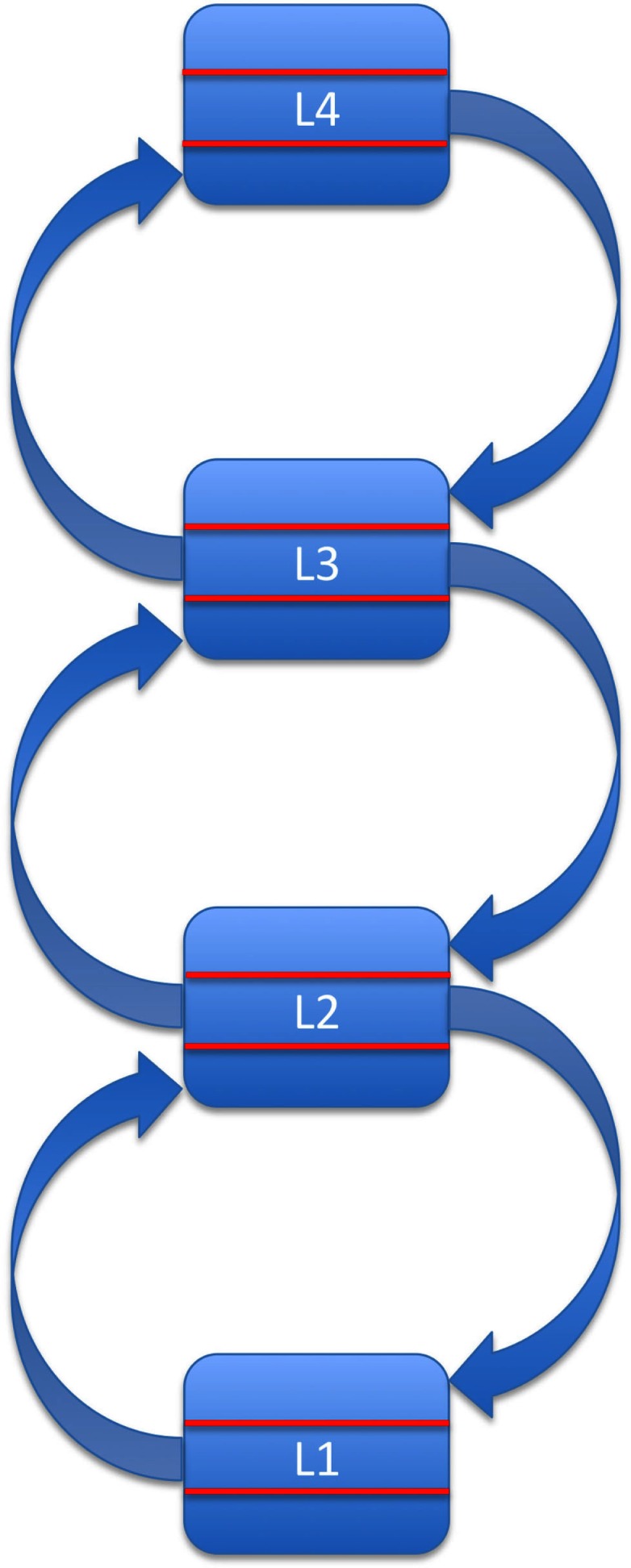
**Bottom-up processing as a series of loops formed by ascending and descending connections between neighboring levels in a hierarchically organized system**. For simplicity, levels L1–L4 are shown as consisting of three layers only (ascending input, descending input, and output).

Combining these assumptions leaves us with a series of loops as shown in Figure 1. We propose that these loops constitute the basis of bottom-up processing. In other words, bottom-up processing consists of one or more cycles of feedforward-feedback activity along ascending and descending connections, instantiated across pairs of neighboring areas in the hierarchy. This iterative, multi-layered process reflects the current, semi-hardwired architecture of the system, which in turn reflects a combination of phylogenetic and ontogenetic information available to the organism.

The crucial point here is that we explicitly assign a role in bottom-up processing to descending connections between neighboring areas in the hierarchy. Bottom-up processing along this series of loops is reliable and relatively fast (Bullier, 2001; Bacon-Mace et al., 2005). However, it is not very flexible.

It is now generally appreciated that even the earliest levels of sensory processing remain malleable throughout life (Neville and Bavelier, 2002; Bavelier et al., 2010). Animal studies have demonstrated that intense training followed by appropriate consolidation can lead to long-lasting modifications of neural responses in primary sensory cortices virtually from the first spike onward (Crist et al., 2001; Li et al., 2004). Recent evidence from human studies points in the same direction (Schwartz et al., 2002; Pourtois et al., 2008; Bao et al., 2010).

This is what we refer to as a semi-hardwired architecture: one that is sufficiently stable to allow for rapid processing of stimuli that have been frequently encountered, either over the course of a species’ evolution or during an individual’s lifespan; and that is at the same time sufficiently flexible to adapt to lasting and pervasive changes in the organism’s environment. However, neural plasticity at the time-scales examined in the above-mentioned studies does not offer enough flexibility to an organism in a competitive and rapidly changing environment.

Thus, at a conceptual level, top-down processes can be conceived of as influences that confer moment-to-moment flexibility onto the semi-hardwired network that at any given time ensures efficient and reliable sensory processing in known environments. This relates to the assumption that the hierarchical organization of sensory systems seen across modalities and species reflects two basic imperatives in terms of predictive coding (Barlow, 1985): first, to take in a maximum of new information in order to detect contingencies in the environment; and second, to exploit these contingencies, once extracted, to construct predictions about the environment which can be rapidly applied to guide adaptive behavior.

Contingencies and the predictions based upon them vary in complexity, and there is rich evidence showing that low-level contingencies may be reflected in the basic organization of low-level sensory cortices (Rao and Ballard, 1999; Barlow, 2001), and even at the level of the retina (Srinivasan et al., 1982; Barlow, 1997; Hosoya et al., 2005). A particularly striking example is given by the seminal work of Rao and Ballard (1999): the authors trained an artificial neural network with natural images and explained end-stopping, an extra-classical receptive field property of V1 neurons, in terms of the frequent occurrence in their stimulus material of oriented lines extending beyond the small receptive fields of the model’s V1 neurons.

High-level contingencies, on the other hand, relate to the higher-order causes of what is currently perceived (Friston, 2005) and can often only be detected by integrating information across large portions of space, long intervals of time, and multiple modalities (Clark, in press). As an example, consider natural languages: understanding a spoken or written phrase in German can be complicated by the fact that the verb may be positioned more flexibly than for example in English. Thus, crucial aspects of the information transmitted remain unspecified for different periods of time, requiring different processes of verification, interpretation, and prediction (Dambacher et al., 2009). Importantly, such differences are completely independent of the actual content of the message and also, to some extent, independent of sensory modality. These higher-order contingencies may be subject to more rapid changes (e.g., switching between languages) than the regularities to which the lower levels of sensory systems are tuned (Kersten et al., 2004), thus rendering adaptations based on long-lasting structural reconfigurations or stimulus-specific functional changes inappropriate.

We assume that the higher levels in our model system extract such higher-order contingencies and dynamically use them to create a set of relatively abstract predictions that can be rapidly adapted or exchanged. However, for these abstract predictions to be useful, there must be a way for them to effectively modulate iterative bottom-up processing at many different levels of the hierarchy. Therefore, we assume that top-down processes include some form of bypassing or short-circuiting of bottom-up processes. The fastest and most direct route to achieve this would be via direct connections between high-level region L(z) and low-level region L(x) which are not hierarchical neighbors and therefore do not form a bottom-up processing loop.

As a first approximation, we thus define top-down processes as instances of direct information transfer from higher to lower regions that skip at least one level in the hierarchy. This anatomical criterion for distinguishing top-down from bottom-up processes specifies the minimal requirements for the conceptual framework outlined above. That is, in order to render bottom-up processing more flexible, high-level regions are assumed to modulate activity in low-level regions using information that is not represented at intermediate levels.

**Figure 2:**
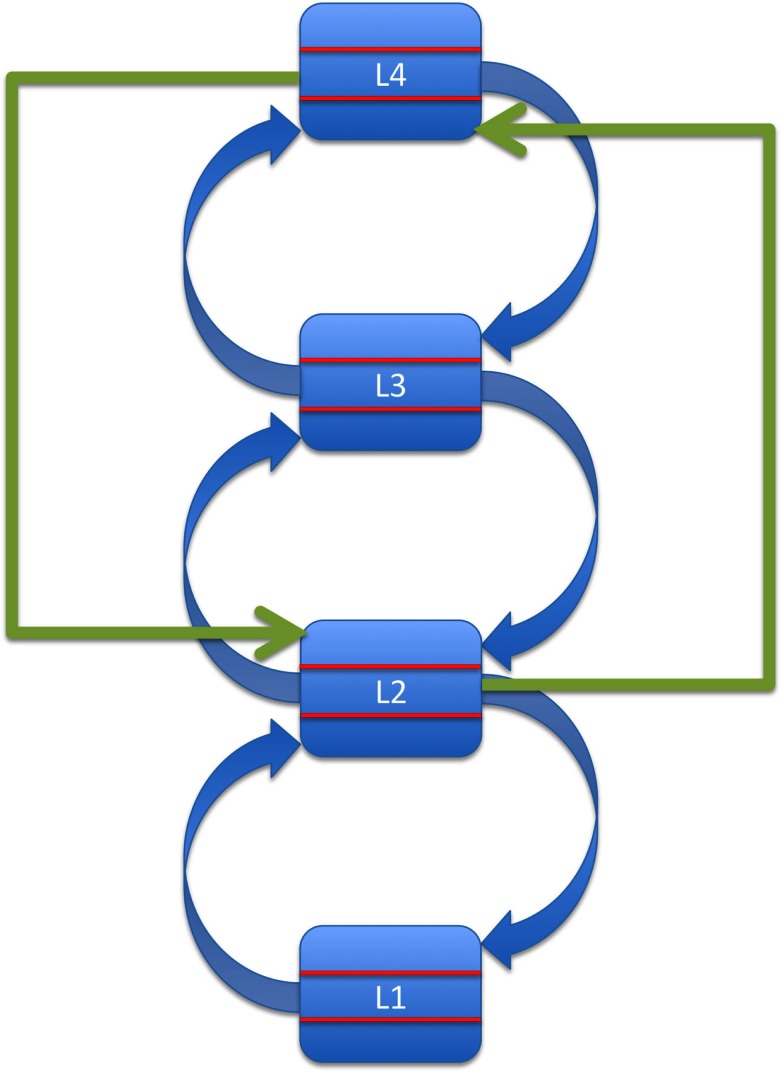
**Top-down effects as direct influences of a source region located at least two levels above the target region in a hierarchical predictive-coding system**.

This basic idea is depicted in Figure 2: a high-level region relays information directly to a lower tier of the system, bypassing an area situated more immediately upstream of this target region. Given the reciprocity of most cortical connections (Felleman and Van Essen, 1991), we assume that top-down processes also involve bidirectional information exchange, i.e., they rely on loops of ascending and descending connections distinct from those mediating bottom-up processing.

The main point here is that in our model, backward information transfer between immediate neighbors in the hierarchy does not qualify as top-down processing. This is important because it separates our view of top-down processes from an apparently simpler one based only on direction of information flow.

In summary, we propose an anatomical criterion for the separation of functional activity patterns into categories of bottom-up and top-down. Given a strictly hierarchical system, this criterion specifies the minimal requirements for classifying a particular pattern of effective connectivity as top-down: namely, there must be a direct anatomical connection between a source and a target region, and the source must be located at least two levels above the target.

### Testing the predictive-coding account

It is important to note that our proposals are not meant to be a comprehensive theory of hierarchical processing. Rather, we provide axiomatic definitions of two equivocal terms commonly invoked to build theories and to interpret empirical results. Our ideas should be critically discussed and empirically tested. Ideally, these tests would demonstrate two things:

1. Our proposals provide a better heuristic for interpreting previous empirical results than current loose notions of bottom-up and top-down processes.
2. Our proposals provide novel hypotheses and correctly predict the outcome of future experiments.

#### Explaining previous results

An important limitation of current descriptions of bottom-up and top-down processes is that they largely fail to explain fundamental effects on perceptual processing, such as priming (Grill-Spector et al., 2006). This limitation clearly emerges in the discussion of the position paper by Theeuwes (2010a) already mentioned. A large part of this discussion focuses on whether priming should be conceived of as a bottom-up (Theeuwes, 2010a,b) or a top-down process (de Fockert, 2010; Egeth et al., 2010; Eimer and Kiss, 2010; Kristjánsson, 2010; Müller et al., 2010).

On the one hand, priming can be seen as a very basic process that one is tempted to classify as purely bottom-up: it is rapid (Dehaene et al., 2001), often automatic in the sense of being unrelated to the observer’s goals (Moors and De Houwer, 2006), and it can apply to very basic stimulus characteristics coded at the lowest levels of sensory processing (Maljkovic and Nakayama, 1994, 1996). On the other hand, an increasing body of evidence shows that priming effects can be object-related (Chun and Jiang, 1998; Kristjansson et al., 2008) as well as linked to the observer’s goals (Kiefer and Martens, 2010). More importantly, it is difficult to describe priming as purely stimulus-driven, as it reflects an effect of previous stimulation, and hence some kind of memory trace has to be involved (Kristjánsson, 2010).

We believe our proposals could prove valuable in resolving this debate. They suggest that priming can be instantiated both along the loops of ascending and descending connections between hierarchical neighbors that we have described as the anatomical basis of bottom-up processing, and via top-down processes that operate along long-range cortico-cortical connections (Summerfield et al., 2006). In this view, priming effects are not seen as a monolithic phenomenon reflecting a unitary process (for a similar arguments, see Henson, 2003; Kristjánsson and Campana, 2010). Rather, different forms of priming may arise depending on the processes and pathways recruited to yield this sensory facilitation. More specifically, discrepancies in the literature concerning the influence of goals, task set, etc., on priming could be linked to the involvement (or not) of high-level control regions that represent task-relevant contingencies.

#### Deriving novel hypotheses

Our proposals rest on the assumption that top-down processes confer flexibility onto the semi-hardwired network underlying bottom-up processes by short-circuiting the latter. One way to test this central assumption is to show that in the absence of top-down input, bottom-up processing is either substantially delayed or fails entirely (Super et al., 2001). As an example for testing this idea, consider a study by Muckli et al. (2005), who showed that the perception of apparent-motion leads to BOLD activity in V1 along the apparent-motion trajectory. The authors concluded that the most likely source for this effect was feedback from area MT to V1.

Given that MT is located several levels above V1 in the visual hierarchy (Felleman and Van Essen, 1991), an unequivocal demonstration that the efficient detection of the (illusory) motion stimulus depends on top-down processes short-circuiting bottom-up processes would require selectively deactivating the connections between V1 and MT in both directions. This should preserve processing along the hierarchy of bottom-up loops up to and beyond MT. Our model suggests that in this case, the perception of illusory motion should be absent, or at least substantially delayed and reduced.

While this experiment may be difficult to carry out with current methods, more realistic approaches exist. For example, in an extension of previous work (Pascual-Leone and Walsh, 2001) using transcranial magnetic stimulation (TMS), one could temporarily deactivate MT, which should not only reduce the perception of apparent motion, but also the concomitant activity along the apparent-motion trajectory in V1. Such an approach cannot isolate the specific contributions of direct connections between MT and V1. However, if similar effects can be obtained for other types of illusions, involving high-level visual areas other than MT, this would offer converging evidence for our view.

## Conclusion

Based on a predictive-coding model, we have outlined a conceptually unequivocal distinction between bottom-up and top-down processes that addresses some of the limitations of our current understanding of these terms. Our proposals highlight the mutual interdependence and constant interaction between bottom-up and top-down processes. Thus, rather than searching for cases of pure bottom-up or top-down processing, future efforts should address their relative contributions as well as the mechanisms of their interaction in the context of a given task.

## Conflict of Interest Statement

The authors declare that the research was conducted in the absence of any commercial or financial relationships that could be construed as a potential conflict of interest.
